# Hidden health IT hazards: a qualitative analysis of clinically meaningful documentation discrepancies at transfer out of the pediatric intensive care unit

**DOI:** 10.1093/jamiaopen/ooz026

**Published:** 2019-08-07

**Authors:** Evan W Orenstein, Daria F Ferro, Christopher P Bonafide, Christopher P Landrigan, Scott Gillespie, Naveen Muthu

**Affiliations:** 1 Department of Pediatrics, Emory University School of Medicine, Atlanta, Georgia, USA; 2 Division of Hospital Medicine, Children’s Healthcare of Atlanta, Atlanta, Georgia, USA; 3 Department of Biomedical and Health Informatics, Children’s Hospital of Philadelphia, Philadelphia, Pennsylvania, USA; 4 Department of Pediatrics, Perelman School of Medicine, University of Pennsylvania, Philadelphia, Pennsylvania, USA; 5 Division of General Pediatrics, Department of Pediatrics, Boston Children’s Hospital, Boston, Massachusetts; 6 Harvard Medical School, Boston, Massachusetts, USA; 7 Division of Sleep and Circadian Disorders, Departments of Medicine and Neurology, Brigham and Women’s Hospital, Boston, Massachusetts, USA

**Keywords:** medical errors, patient handoff, electronic health records, hospital communication systems

## Abstract

**Objective:**

The risk of medical errors increases upon transfer out of the intensive care unit (ICU). Discrepancies in the documented care plan between notes at the time of transfer may contribute to communication errors. We sought to determine the frequency of clinically meaningful discrepancies in the documented care plan for patients transferred from the pediatric ICU to the medical wards and identified risk factors.

**Materials and Methods:**

Two physician reviewers independently compared the transfer note and handoff document of 50 randomly selected transfers. Clinically meaningful discrepancies in the care plan between these two documents were identified using a coding procedure adapted from healthcare failure mode and effects analysis. We assessed the influence of risk factors via multivariable regression.

**Results:**

We identified 34 clinically meaningful discrepancies in 50 patient transfers. Fourteen transfers (28%) had ≥1 discrepancy, and ≥2 were present in 7 transfers (14%). The most common discrepancy categories were differences in situational awareness notifications and documented current therapy. Transfers with handoff document length in the top quartile had 10.6 (95% CI: 1.2–90.2) times more predicted discrepancies than transfers with handoff length in the bottom quartile. Patients receiving more medications in the 24 hours prior to transfer had higher discrepancy counts, with each additional medication increasing the predicted number of discrepancies by 17% (95% CI: 6%–29%).

**Conclusion:**

Clinically meaningful discrepancies in the documented care plan pose legitimate safety concerns and are common at the time of transfer out of the ICU among complex patients.

## BACKGROUND AND SIGNIFICANCE

Handoffs are formal transitions in responsibility and information between providers caring for a patient provided verbally, in writing, or both. Despite major advances in handoff processes, communication failures leading to medical errors remain an important cause of harm among hospitalized patients. These medical errors are often preceded by miscommunications during handoffs, which can be compounded by inefficient documentation systems.^1^^,^^2^ In a series of 197 perioperative safety incidents, inadequate documentation was identified as the most common cause of communication breakdown.^3^ Conversely, accurate structured handoff documents that support verbal communications are associated with improved outcomes.^4–7^ Despite the importance of accurate documentation for communication, handoffs often contain discordant information when compared to other parts of the medical record for the same patient at the same time.^8–10^ The architecture and communication goals of the care plan in the handoff differ from those in clinician notes, with handoff documents generally focused solely on provider–provider communication where transfer notes frequently contain additional information for regulatory purposes. Providers must update these documents independently, which is time consuming, and may be inefficient and error prone. Frequent updates of the care plan across multiple documents is likely to lead to lower quality handoffs as well as discrepancies—factual inconsistencies in the patient’s care plan across documents.^8^^,^^11–13^ While information seeking patterns of individual providers or teams may vary, the mere existence of discrepant documents increases the risk of misinterpretation that can lead to uncertainty and subsequent error.^2^

However, the frequency, clinical significance, and contribution of documentation discrepancies in the care plan to medical errors remain unknown. In part, this gap is due to difficulty measuring and classifying meaningful discrepancies. Many important elements are communicated via free text and require clinical and contextual knowledge to evaluate their significance. To our knowledge, no studies have evaluated the frequency and severity of documentation discrepancies in the overall care plan.

The risk of medical errors increases during transfers from the intensive care unit (ICU) to the wards, frequently due to communication errors.^1^^,^^14^ The presence of documentation discrepancies at this vulnerable point in patient care may cause communication failures that lead to medical errors. In this study, we aimed to determine the frequency of clinically meaningful discrepancies for patients transferred out of the pediatric ICU to medical wards; to classify discrepancy types; and to identify risk factors for clinically meaningful discrepancies at the time of transfer.

## MATERIALS AND METHODS

### Study setting, population, and document identification

Children’s Hospital of Philadelphia is a free-standing pediatric hospital that serves both as a quaternary, academic referral center for complex patients and as the community hospital for West and Southwest Philadelphia. Hospital policy dictates that all patients transferred from the pediatric ICU to medical wards should have a transfer note signed prior to the time of transfer to communicate the hospital course and current care plan to the receiving team. In addition, it is standard practice for clinicians in the pediatric ICU and on medical wards to maintain a wiki-style handoff in the vendor (Epic Systems©) electronic health record (EHR), with the written handoff structured according to I-PASS guidelines.^4^ At the time of transfer out of the pediatric ICU, both these documents should reflect the same care plan as responsibility is formally transferred from one primary team to another. Although the format may appropriately differ to accommodate the different audiences and goals of each document, there is no mechanism to ensure alignment of the content between the handoff and progress/transfer notes other than providers’ manual updates.

We identified all patients who had been admitted to the pediatric ICU for at least 24 hours at Children’s Hospital of Philadelphia and were subsequently transferred to medical wards between January 1, 2017 and December 31, 2017. We excluded patient transfers without a signed transfer note in the EHR prior to or up to 2 hours after the transfer, using the location time stamp in the EHR. While individual patients could have multiple transfers out of the pediatric ICU during a single hospital stay, we only evaluated documents from 1 transfer per patient.

For each transfer evaluated, two physician reviewers (EWO and DFF) compared the transfer note and handoff document. Both EWO and DFF had experience caring for patients transferred from the pediatric ICU to medical wards. The reviewers preferentially selected the closest signed transfer note up to the time of transfer. However, if no transfer note was signed prior to transfer, we selected the first signed version up to 2 hours after the time of transfer. We extracted the most recent update to the handoff document prior to the time of transfer. To assess for risk factors for clinically meaningful discrepancies, we collected the patient’s age, gender, total length of stay in the hospital, and length of ICU stay all at the time of transfer out of the pediatric ICU. We also counted the number of unique medications the patient received in the 24 hours prior to transfer out. The length in characters of the full transfer note, transfer note assessment & plan only, and the handoff note were also recorded.

### Coding procedure

**Figure 1.**
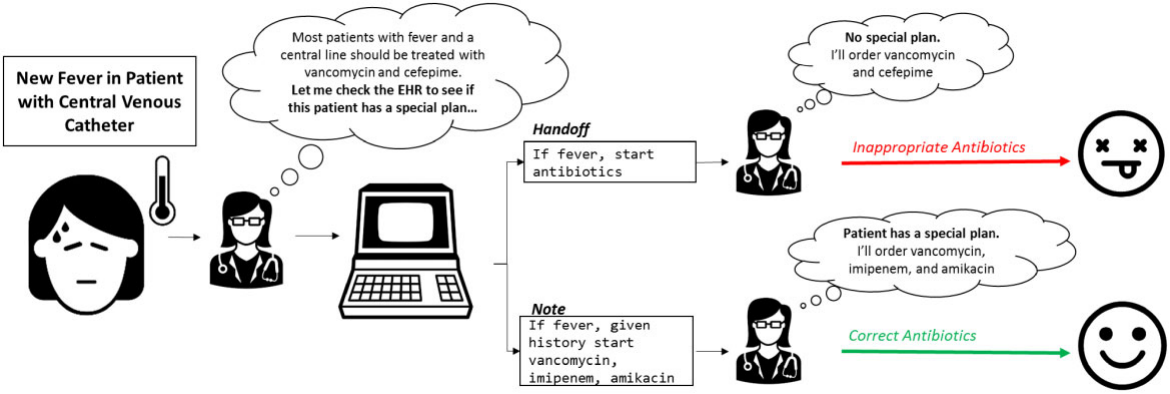
Conceptual model of documentation discrepancies leading to serious medical error.

Similar to previous studies examining documentation discrepancies,^15^ clinically meaningful discrepancies were defined as discrepancies in which: “if a physician saw the information only from one document (i.e., the handoff only or the transfer note only), it is plausible to make a decision that could lead to severe patient harm because of inaccurate or incomplete information.” This definition was based on the principle that in the setting of clinical uncertainty, providers often satisfice,^16^ consuming just enough information to justify a clinical decision, even when further investigation might reveal that decision to be inappropriate (Figure 1).

**Figure 2.**
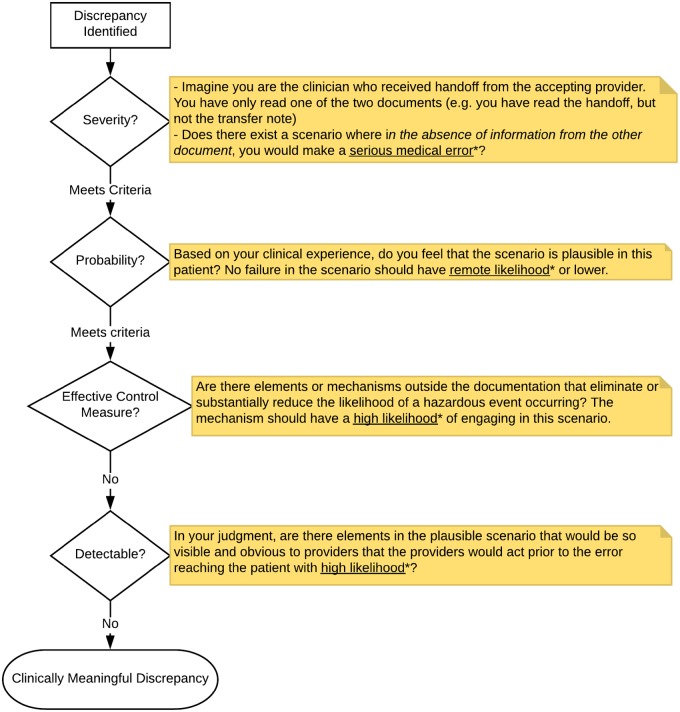
Algorithm to determine if documentation discrepancy constitutes a clinically meaningful discrepancy. After detecting a clinically meaningful discrepancy, reviewers were instructed to determine if a clinical decision based on one of the documents in the absence of information from the other could lead to a serious medical error.^18^ Serious medical errors were defined using the National Coordinating Council for Medication Error Reporting and Prevention category F (prolongs hospitalization), G (requires life-saving intervention), or H (results in permanent harm).^19^ Reviewers then enumerated all process failures required for severe harm and discarded discrepancies where any process failure had remote likelihood or lower.^20^ The reviewer then assessed if there existed mechanisms outside documentation that would eliminate or substantially reduce the likelihood of the error reaching the patient^21^ such as an allergy warning. Finally, the reviewer determined if elements in the scenario would be so visible and obvious that providers would act to prevent the error with high probability. See appendix for examples.

To operationalize this definition, we adapted the Healthcare Failure Mode and Effect Analysis (HFMEA) framework, which helps multidisciplinary teams proactively evaluate vulnerabilities in patient safety.^17^ Specifically, HFMEA requires a study team to explicitly identify all the failures in a process that could lead to a patient safety event, to score each failure in terms of its severity and probability, and to determine if existing control measures would prevent the error from reaching the patient. The HFMEA severity and probability scales were adapted to focus on medical errors for individual patients (Figure 2). The reviewer then categorized the information type of each clinically meaningful discrepancy based on a taxonomy developed iteratively during coding procedure development, informed by prior handoff frameworks.^11^^,^^22^ Finally, we classified the source of the discrepancy as a transfer note omission (information in the handoff document missing from the transfer note), handoff document omission (information in the transfer note missing from the handoff document), or direct contradiction (discrepant information between the documents about the same information element). Further details about the coding procedure including example discrepancies are described in the Supplementary Appendix.

### Data analysis

During our study period, 900 patients were transferred from the pediatric ICU to medical wards, of whom 782 had a signed transfer note prior to or up to 2 hours after the transfer location time stamp in the EHR. We examined 11 transfers during coding procedure development. We randomly selected 50 transfers for independent evaluation by 2 physician - informaticists (EWO and DFF) to assess inter-rater reliability. We primarily assessed the agreement of reviewer ratings for the number of clinically meaningful discrepancies in each transfer using two-way mixed, absolute agreement, single-measures intraclass correlation (ICC). We also assessed agreement for the presence of ≥1 clinically meaningful discrepancy in each transfer using Cohen’s Kappa. In classifying the clinical meaningfulness of individual discrepancies, reviewers each provided individual assessments and differences were resolved by consensus. Inter-rater reliability calculations were performed in R version 3.3.3^23^ using the irr^24^ package.

We performed multivariable regression using negative binomial models to assess the influence of risk factors on the number of clinically meaningful discrepancies detected by consensus. We evaluated the influence on the number of clinically meaningful discrepancies of age, sex, length of stay, transfer note and handoff length (restricting to assessment and plan sections), and the number of unique medications the patient received in the 24 hours prior to transfer as a marker of complexity. We initially performed univariate analysis, and variables with *P* value < 0.2 in univariate analysis were considered for the multivariable model. Model selection was performed using forward selection based on significant bivariate results. To evaluate for possible overfitting given the sample size, we performed a sensitivity analysis comparing the results of negative binomial regression for the count outcome (number of clinically meaningful discrepancies) to a more conservative logistic regression model using a dichotomous outcome (presence or absence of clinically meaningful discrepancies). We used SAS software v.9.4 (Cary, NC)^25^ for regression analyses.

### Protection of human subjects

This study was approved by the Institutional Review Board of Children’s Hospital of Philadelphia.

## RESULTS

A total of 34 clinically meaningful discrepancies were identified by consensus among the 50 patient transfers, yielding a mean of 0.68 ± 0.22 per transfer (Table 1). At least 1 clinically meaningful discrepancy was identified (range: 1–8) between the transfer note and handoff document in 14 transfers (28%), and at least 2 clinically meaningful discrepancies were present in 7 transfers (14%). Most clinically meaningful discrepancies were clustered in a small number of transfers; only 4 transfers had 3 or more clinically meaningful discrepancies, but the total of 21 discrepancies observed in these 4 transfers represented over half of all clinically meaningful discrepancies observed in the study. The most common source of clinically meaningful discrepancies was transfer note omissions (*n* = 20, 59%) followed by direct contradictions between the documents (*n* = 10, 29%). Only 4 (12%) discrepancies were due to handoff omissions.

**Table 1.** Number of CMDs detected

# CMDs per transfer	Number of transfers (%)
0	36 (72)
1	7 (14)
2	3 (6)
3	1 (2)
4	1 (2)
5	0 (0)
6	1 (2)
7	0 (0)
8	1 (2)
Total	50 (100)

CMDs: clinically meaningful discrepancies.

We classified each clinically meaningful discrepancy into a taxonomy of clinical communication elements (Table 2). The most frequent categories were discrepancies in situation awareness notifications (62%), current therapy (38%), and important historical elements (18%). Many clinically meaningful discrepancies involved multiple categories. In particular, all four discrepancies where an important problem was missing or represented differently across documents also had a discrepancy in current therapy or a situation awareness notification.

**Table 2.** Categories and examples of clinically meaningful discrepancies

Category	*N* (%)	Example discrepancy	Potential harm scenario
Situation Awareness	21 (62)	*Handoff:* “^a^Mitochondrial Disease: AVOID steroids, LR, or high dextrose fluids” *Transfer note:* No mention of medications to avoid.	If receiver not aware of medication restrictions, they may order steroids, lactated ringers, and/or high dextrose fluids as these are commonly prescribed. This could easily lead to acidosis in this patient with mitochondrial disease, prolonging hospitalization, or worse.
Current therapy	13 (38)	*Handoff:* “bactrim ppx/valium (HOLD)” *Transfer note:* “continue bactrim ppx and valium”	Receiver will likely choose to hold or give bactrim (trimethoprim/sulfamethoxazole) and valium (diazepam) based on which document they read. Inappropriately holding these medications could lead to unnecessary bladder spasm and infection. Inappropriately giving these medications could lead to reaction from unnecessary antibiotic or oversedation. Either of these could prolong hospitalization or worse.
History	6 (18)	*Handoff:* “^a^History of ventricular arrhythmias and on multiple QT prolonging medications, history of Q wave on ECG” *Transfer Note:* No mention of this history or problem.	If receiver not aware of high risk from QT prolongation, very likely would prescribe frequently used QT-prolonging medications at some point in hospitalization, which could lead to arrhythmia, leading to life-threatening or permanent harm.
Problems	4 (12)	*Handoff:* “#FEN: Hyponatremia”—no mention of free water flushes *Transfer Note:* “Resolving hypernatremia – continue increased free water flushes”	If receiver thinks patient currently has hyponatremia, may hold or discontinue free water flushes. If receiver thinks patient has hypernatremia, will likely continue free water flushes. Inappropriate decision could lead to electrolyte disturbances that prolong hospitalization or worse.
Action Item	2 (6)	*Handoff:* “[ ] wound c/s re central line related erythema/swelling” *Transfer note:* No mention of the action item; exam states “Broviac site c/d/i”	If receiver not aware of erythema/swelling at central line site and of action item to consult wound care, patient may develop preventable central-line associated bloodstream infection, which could prolong hospitalization or worse.

^a^The percentages add up to more than 100 because 1 clinically meaningful discrepancy could fit multiple categories.

### Inter-rater reliability

The two reviewers (DFF and EWO) identified 21 and 46 clinically meaningful discrepancies each, of which 14 were identified by both reviewers. Twenty clinically meaningful discrepancies were identified by only one reviewer but accepted as clinically meaningful by the second reviewer when brought up in consensus discussion. Nineteen discrepancies were initially deemed clinically meaningful by one reviewer but felt to not meet the definition during consensus discussion. The overall ICC for the absolute number detected by the two reviewers was moderate at 0.70 (95% CI: 0.41–0.84). Cohen’s kappa for the presence of ≥1 discrepancy was fair at 0.43.

### Risk factors

We evaluated the association between the number of clinically meaningful discrepancies with demographic variables, document length, length of stay, and the number of active medications at time of transfer in both univariable (Table 3) and multivariable (Table 4) analyses. In univariable analysis, all independent variables except age and gender were significant predictors of the number of clinically meaningful discrepancies, with the largest effect sizes from longer handoff document length, longer transfer note assessment and plan length, and more unique medication routes in the prior 24 hours. Having handoff note length in the top quartile (>2500 characters) increased the expected number of clinically meaningful discrepancies by the largest factor, 45-fold higher (95% CI: 5.47–370.00) when compared to those with handoff length <1500 characters. Those with handoff notes >2500 characters were predicted to have 2.25 (95% CI: 1.17–4.32) clinically meaningful discrepancies. Each increase by 1 of the number of unique medication-routes the patient received in the 24 hours prior to transfer multiplied the predicted number of clinically meaningful discrepancies by 1.30 (95% CI: 1.09–1.54); transfers with >9 unique medication-routes administered in the 24 hours prior to transfer had 13.15 (95% CI: 3.28–52.69) times more clinically meaningful discrepancies than transfers with ≤9 such administrations. In a sensitivity analysis using logistic regression, we found similar relationships and preservation of statistical significance between independent variables and the presence or absence of ≥1 clinically meaningful discrepancy in each transfer, with the exception of the PICU and hospital length of stay exposures (Supplementary Appendix Table A3).

**Table 3.** Contribution of independent variables to the number of clinically meaningful discrepancies between the transfer note and handoff note: univariate analysis

Characteristic, *N* (%)	Summary *N* = 50	CMDs *N* = 34	Rate ratio (95% CI)	*P* value
Age (years)				
>11 years	13 (26%)	14 (41%)	4.85 (0.56, 42.30)	0.153
5–11 years	16 (32%)	8 (24%)	2.25 (0.26, 19.71)	0.464
1–4 years	12 (24%)	10 (29%)	3.75 (0.41, 34.23)	0.241
<1 years	9 (18%)	2 (6%)	Reference	–
Gender				
Female	27 (54%)	22 (65%)	1.56 (0.42, 5.77)	0.504
Male	23 (46%)	12 (35%)	Reference	–
PICU LOS				
≥5 days	11 (22%)	19 (56%)	4.49 (1.22, 16.50)	**0.024**
<5 days	39 (78%)	15 (44%)	Reference	
Hospital LOS				
≥ 5 days	16 (32%)	26 (76%)	6.91 (2.17, 21.96)	**0.001**
< 5 days	34 (68%)	8 (24%)	Reference	
Unique medication-routes^a^ in 24 h prior to transfer				
>9	22 (44%)	31 (91%)	13.15 (3.28–52.69)	**<0.001**
≤9	28 (56%)	3 (9%)	Reference	
Transfer note assessment and plan length				
>1500 characters	12 (24%)	25 (73%)	18.75 (3.51, 100.26)	**0.001**
750–1500 characters	20 (40%)	7 (21%)	3.15 (0.55, 17.95)	0.196
<750 characters	18 (36%)	2 (6%)	Reference	–
Handoff document length				
>2500 characters	12 (24%)	27 (79%)	45.00 (5.47, 370.00)	**<0.001**
1500–2500 characters	18 (36%)	6 (18%)	6.67 (0.74, 60.19)	0.091
<1500 characters	20 (40%)	1 (3%)	Reference	–

CMDs: Clinically meaningful discrepancies.

^a^For example, administration of IV ranitidine and PO ranitidine in the 24 hours prior to transfer count as 2 unique medication routes. By contrast, a PO administration of 75 mg of ranitidine at 1 time and 150 mg of ranitidine at a different time count as only 1 unique medication route.

**Table 4.** Contribution of independent variables to the number of clinically meaningful discrepancies between the transfer note and handoff note: multivariable analysis

Characteristic, *N* (%)	Rate ratio (95% CI)	*P* value
Hospital LOS		
≥5 days	3.05 (1.30, 7.21)	**0.011**
<5 days	Reference	
Unique medication-routes^a^ in Prior 24 h	1.17 (1.06, 1.29)	**0.002**
Handoff document length		
>2500 characters	10.59 (1.24, 90.18)	**0.031**
1500–2500 characters	1.82 (0.19, 17.10)	0.602
<1500 characters	Reference	

^a^For example, administration of IV ranitidine and PO ranitidine in the 24 hours prior to transfer count as 2 unique medication routes. By contrast, a PO administration of 75 mg of ranitidine at 1 time and 150 mg of ranitidine at a different time count as only 1 unique medication route. Bold numbers signify statistical significance (*P* < 0.05).

The final multivariable model included the length of stay in the hospital prior to transfer (which may include multiple pediatric ICU stays), the number of unique medication routes in the prior 24 hours, and the handoff document length; all other variables fell out of the model using the forward selection process described above. Handoff document length >2500 characters (top quartile) and number of unique medication-routes the patient received in the 24 hours prior to transfer had the greatest influence on the number of clinically meaningful discrepancies, although hospital length of stay prior to transfer remained a significant independent predictor.

## DISCUSSION

In our analysis of transfer notes and handoff documents for 50 patients transferred from the pediatric ICU to medical wards, 14 transfers (28%) had at least one discrepancy between the two documents determined by two physician reviewers to be high risk of causing a communication failure leading to a severe medical error. Seven transfers (14%) had at least two clinically meaningful discrepancies between the transfer note and handoff documents. The most common information categories included discrepancies in situational awareness notifications and current therapy for the patient, and the most common source of discrepancies was transfer note omissions. We hypothesize that the extra work required to keep the care plan updated in multiple documents in a highly interruptive context with little downtime likely contributes to the clinically meaningful discrepancies seen in this study. Since handoffs occurred on a daily basis while transfers out of the ICU were less frequent, we suspect that providers updated handoff documents more often but frequently omitted key information or failed to cross-reference when hurriedly editing the transfer note for complex patients just prior to transfer. In the absence of a system to harmonize the many documents that inpatient providers must keep updated, errors with potential serious clinical significance are common.

Simply introducing computerized handoff tools that mirror paper counterparts may not improve provider communication. In the first study of medical errors following implementation of a resident handoff bundle,^26^ the addition of a computerized handoff tool did not lead to a greater reduction in medical errors than educational and environmental interventions alone. Transition from a paper handoff to an electronic handoff tool also did not reduce the number of discrepancies in verbal handoffs in a study of nurses, residents, and fellows in a pediatric ICU.^27^ Thus, handoff documentation systems that are disconnected from the care plan in other areas of the EHR may not prevent communication errors as effectively as intended. By contrast, care coordination and handoff tools that store discrete data for problems, tasks, and situation awareness and reorganize those data into different views for different contexts of information use have the potential to reduce medical errors, and may lead to shorter length of stay and reduced readmission rates.^28^

To our knowledge, no studies have examined the potential for error associated with discrepancies in free text in the care plan. Previous studies have estimated the potential for harm from discrepancies in medication instructions and demonstrated inter-rater reliability between subject matter experts through iteratively developed rule-based algorithms.^15^^,^^29^ Automated natural language processing approaches to detect these types of discrepancies have been developed in the medication order context,^30^ however, this problem may be more complex when applied to a more general care plan.

Our study has several important limitations. Determining the importance of discrepancies in the documented care plan is particularly challenging since the significance is dependent on the clinical context, the knowledge of the reviewer, and the assumed knowledge of receivers of the documents. Similarly, consistently identifying the same discrepancies between two large, dense documents is difficult with long and complicated care plans described in handoffs and transfer notes. The two reviewers frequently identified different discrepancies, requiring discussion to come to consensus. Thus, discrepancy review by an individual physician is not sufficiently reliable even aided by a standard algorithm. Ensuring appropriate identification and classification of discrepancies likely requires multiple reviewers with substantial expertise including medical and contextual knowledge, a resource-intensive process. In addition, we have not yet established an association between the presence of clinically meaningful discrepancies at the time of transfer out of the ICU and subsequent serious medical errors. Given the association with prior length of stay and the length of the handoff document, these discrepancies may be a marker of complexity rather than an independent cause of subsequent errors. Alternatively, longer handoffs may be associated with copy-pasted or auto-populated content.^31^ Reorganizing notes and handoffs into a single source of truth focused on key elements of communication such as problems, situation awareness, and tasks would likely reduce the documentation burden, minimize the risks of discrepancies, and reduce information overload for handoff recipients.

## CONCLUSIONS

Structured communication strategies during transfer of care have demonstrated reductions in medical errors and preventable adverse events. However, documentation systems to support this communication currently require redundant efforts from providers, leading to poorly updated written plans of care and clinically meaningful discrepancies. Future work to determine if these discrepancies cause subsequent errors would strengthen the case for more streamlined documentation strategies. Human factors engineering approaches to harmonize documentation for handoffs with regulatory and institutional requirements for the formal medical record may improve patient safety in addition to reducing clinicians’ documentation burden.

## SUPPLEMENTARY MATERIAL

Supplementary material is available at Journal of the American Medical Informatics Association online.

## AUTHOR CONTRIBUTIONS

EWO, DFF, CPB, CPL, and NM designed the study.

EWO, DFF, and NM performed data collection.

EWO and SG performed the statistical analysis.

EWO, DFF, CPB, CPL, SG, and NM wrote the manuscript.

## CONFLICTS OF INTEREST

EWO, DFF, and NM have equity in Phrase Health©, a healthcare governance and analytics company.

CPL consulted with and holds equity in the I-PASS Patient Safety Institute, a company that seeks to train institutions in best handoff practices and aid in their implementation. He has also received monetary awards, honoraria, and travel reimbursement from multiple academic and professional organizations for teaching and consulting on sleep deprivation, physician performance, handoffs, and safety, and has served as an expert witness in cases regarding patient safety and sleep deprivation.

## Supplementary Material

ooz026_Supplementary_Data
