# Supplementary material for: Hidden health IT hazards: a qualitative analysis of clinically meaningful documentation discrepancies at transfer out of the pediatric intensive care unit
Source: JAMIA Open. 2019 Aug 7;2(3):392–8. doi: 10.1093/jamiaopen/ooz026 (PMC6951953; doi:10.1093/jamiaopen/ooz026)
Supplement: ooz026_Supplementary_Data [file ooz026_supplementary_data.docx]

# APPENDIX

**Probability Scales for the Coding Procedure**

*Probability.* Each step in the process failure leading to severe patient harm identified by the reviewer must have at least occasional likelihood in the experience of the reviewer, based on the following scale from Glaab et al^22^ as defined in Table A1:

**Table A1:** Probability scale for determining if a risk is sufficiently *probable* to be a clinically meaningful discrepancy.

| Extremely Unlikely | Virtually impossible or no known occurrences on similar products or processes, with many running hours. |
| --- | --- |
| Remote | Relatively few failures on similar products or processes, with many running hours. |
| Occasional | Occasional failures on similar products or processes. |
| Reasonably Possible | Repeated failures on similar products or processes. |
| Frequent | Failure is almost inevitable |

*Effective Control Measures.* Elements or mechanisms outside the documentation that eliminates or substantially reduces the likelihood of a hazardous event occurring. The mechanism must engage with high likelihood, defined as in Table A2:^23^

**Table A2:** Probability scale for *effective control measures* and *detectability*.

| Very high | Error almost always detected; or we’ll catch it 9 out of 10 times. |
| --- | --- |
| High | Error likely to be detected; or we’ll catch it 7 out of 10 times. |
| Moderate | Moderate likelihood of detection; or we’ll catch it 5 out of 10 times. |
| Low | Low likelihood of detection; or we’ll catch it 2 out of 10 times |
| Remote | Detection not possible at any point; or we’ll never catch it (0 out of 10 times) |

*Detectability.* Elements in the scenario that would be so obvious that providers would act to prevent the error from reaching the patient with high likelihood, defined as in Table A2.

**Coding Procedure for Example Discrepancies**

*Clinically Meaningful Discrepancy – Example 1:* The handoff note for a patient with a central venous catheter says “if fever, start antibiotics.” The transfer note for the same patient says “if fever, given history of resistant organisms, start vancomycin, imipenem, and amikacin.” The reviewer may envision a scenario where the patient develops a fever due to a brewing central line-associated bloodstream infection with a resistant organism. The provider that looks only at the handoff note orders vancomycin and cefepime (the usual hospital protocol), and the patient’s condition deteriorates as they are not on effective antimicrobial therapy for their resistant organism.

This scenario is considered a clinically meaningful discrepancy because none of the steps in this scenario are of remote likelihood and there is no effective control measure or high likelihood of detectability. Patients with central venous catheter frequently develop central line-associated bloodstream infections. In those previously colonized with resistant organism, there is a high risk that these infections are due to a resistant organism. Providers who are unaware of this history are very likely to order the antibiotics from the hospital standard protocol for fever in patients with a central venous catheter. Patients with a central line-associated bloodstream infection that is being inadequately covered by antibiotics frequently deteriorate, leading to prolonged hospitalization or worse.

*Clinically Meaningful Discrepancy – Example 2:* The transfer note says that if the patient is off their total parenteral nutrition (TPN) for whatever reason, they need Dextrose 7.8% (D7.8) fluids, whereas in the handoff it states they have a history of hypoglycemia and should be started on Dextrose 10% (D10) if TPN falls off and they should receive a D10 bolus if blood glucose falls below 70. The reviewer may envision a scenario where the patient’s TPN is stopped, the provider looks only at the transfer note and orders D7.8 fluids, it takes the pharmacy a long time to mix that custom fluid, the patient is on no dextrose-containing fluids in the meantime and becomes seriously hypoglycemic which could cause permanent harm (NCC MERP Category H).

This scenario is considered a clinically meaningful discrepancy in this patient because none of the steps unrelated to the discrepancy itself in this scenario are of remote likelihood and there is no effective control measure or high likelihood of detectability. It is frequent that TPN must be stopped (e.g. electrolytes in TPN are no longer appropriate, TPN bag spills, TPN is not compatible with other drugs and the patient has limited intravenous access). It is at least occasional that it takes the pharmacy multiple hours to create custom fluids (i.e. not D5 or D10). It is at least occasional that patients who are on TPN 24 hours a day whose TPN is suddenly stopped become hypoglycemic quickly.

*Not a Clinically Meaningful Discrepancy:* The patient is on their home Bilevel Positive Airway Pressure (BiPAP) settings of 15/6 with a rate of 10 while asleep and sprints to room air while awake. The handoff note states that the patient has a history of central apnea, but this information is missing from the transfer note. The reviewer may envision a scenario where a provider eliminates the rate from the BiPAP order and the patient becomes apneic requiring life-sustaining interventions (NCC MERP category G or H).

This scenario is not considered a clinically meaningful discrepancy in this patient because the scenario requires a step unrelated to the discrepancy itself that is of remote likelihood. Specifically, it is of remote likelihood that providers reduce respiratory support below home settings, and when they do it is extremely unlikely that they do not seek more information in the chart and discuss with the specialists who manage that respiratory support long-term.

## Effective Control Measures

### Allergies: When there exists a plausible scenario for harm where in the absence of information from one document or section, the provider would order a medication that they shouldn’t order (e.g. history of paradoxical reaction, red man’s, or another allergy), but that medication is on the patient’s allergy list at the time of ICU transfer, then that counts as an effective control measure and should not be included as a potential error.

*Labs/Results*: When there exists a plausible scenario for harm where in the absence of information from one document or section, the provider might not realize the need to follow up on a particular result (e.g. “f/u blood culture”):

- If the abnormal result would lead to a *phone call* (e.g. blood culture growing) within the institution, this *should count* as an effective control measure of *high* likelihood of engaging. This depends on the lab/image/result being in the same institution; e.g. if a blood culture was drawn at an outside hospital (OSH), this alone would *not* count as an effective control measure if one document said “f/u OSH blood culture” and the other did not.
- If the abnormal result would not lead to a phone call but would lead to a result flag *in time to prevent a potential error*:
  - If the turnaround time is generally less than 24 hours, is run every day, and is ordered frequently in the judgment of the reviewer, then this *should count* as an effective control measure as it is assumed to be of *high* likelihood of engaging.
    - Ex. “f/u electrolytes”; “f/u CBC”; “f/u rapid respiratory viral panel”; “f/u CXR”
  - If the turnaround time is generally >24 hours, is not run every day, or is rarely ordered in the judgment of the reviewer, then this should *not* count as an effective control measure as it is assumed to be of *moderate* likelihood of engaging or lower.
    - Ex. “f/u Urine culture” (generally > 24 hours), “f/u TSH” (may not be run every day), “f/u genetic test” (generally > 24 hours).
- If the abnormal result would not lead to a flag or phone call, this should *not* count as an effective control measure.

## Risk Factors for Clinically Meaningful Discrepancies: Sensitivity Analysis

Due to the small sample size of 50 transfers reviewed, we compared the negative binomial regression model for clinically meaningful discrepancy counts with a binary logistic regression model for the presence of ≥1 clinically meaningful discrepancy. Results were similar for all independent variables except PICU and hospital length of stay. In the negative binomial model, the predicted number of clinically meaningful discrepancies was significantly higher when patients had been in the PICU ≥5 days or in the hospital ≥5 days. In the logistic model, PICU length of stay was not a significant predictor of the presence of at least one clinically meaningful discrepancy, while hospital length of stay was marginally significant.

**Table A3:** Comparison of univariable analysis between negative binomial model with outcome of clinically meaningful discrepancy counts and logistic model with binary outcome of presence/absence of clinically meaningful discrepancies.

|  | **Negative Binomial Model** | | **Logistic Model** | |
| --- | --- | --- | --- | --- |
| **Characteristic, N (%)** | **Rate Ratio**  **(95% CI)** | **P-Value** | **Odds Ratio**  **(95% CI)** | **P-Value** |
| Age (years) |  |  |  |  |
| > 11 years | 4.85 (0.56, 42.30) | 0.153 | 5.00 (0.47, 52.93) | 0.181 |
| 5 – 11 years | 2.25 (0.26, 19.71) | 0.464 | 2.67 (0.25, 28.42) | 0.417 |
| 1 – 4 years | 3.75 (0.41, 34.23) | 0.241 | 4.00 (0.36, 44.09) | 0.258 |
| < 1 years | Reference | -- | Reference | -- |
|  |  |  |  |  |
| Gender |  |  |  |  |
| Female | 1.56 (0.42, 5.77) | 0.504 | 1.19 (0.34, 4.14) | 0.781 |
| Male | Reference | -- | Reference | -- |
|  |  |  |  |  |
| PICU LOS |  |  |  |  |
| ≥ 5 days | 4.49 (1.22, 16.50) | **0.024** | 1.66 (0.40, 6.88) | 0.487 |
| < 5 days | Reference |  | Reference | -- |
|  |  |  |  |  |
| Hospital LOS |  |  |  |  |
| ≥ 5 days | 6.91 (2.17, 21.96) | **0.001** | 3.00 (0.83, 10.91) | **0.095** |
| < 5 days | Reference |  | Reference | -- |
|  |  |  |  |  |
| Unique Medication-Routes* in 24 Hours Prior to Transfer |  |  |  |  |
| > 9 | 13.15 (3.28 – 52.69) | **<0.001** | 7.64 (1.71 – 34.12) | **0.008** |
| ≤ 9 | Reference |  | Reference |  |
|  |  |  |  |  |
| Transfer Note Assessment and Plan Length |  |  |  |  |
| > 1500 characters | 18.75 (3.51, 100.26) | **0.001** | 11.20 (1.74, 72.26) | **0.011** |
| 750 – 1500 characters | 3.15 (0.55, 17.95) | 0.196 | 2.67 (0.45, 15.88) | 0.282 |
| < 750 characters | Reference | -- | Reference | -- |
|  |  |  |  |  |
| Handoff Document Length |  |  |  |  |
| > 2500 characters | 45.00 (5.47, 370.00) | **<0.001** | 37.99 (3.65, 395.03) | **0.002** |
| 1500 – 2500 characters | 6.67 (0.74, 60.19) | 0.091 | 7.31 (0.76, 69.99) | 0.085 |
| < 1500 characters | Reference | -- | Reference | -- |
